# Transitional gradation and the distinction between episodic and semantic memory

**DOI:** 10.1098/rstb.2023.0407

**Published:** 2024-09-16

**Authors:** Hunter Gentry, Cameron Buckner

**Affiliations:** ^1^ Philosophy, Kansas State University, Manhattan, KS 66506, USA; ^2^ Philosophy, University of Florida, Gainesville, FL 32611, USA

**Keywords:** episodic memory, semantic memory, natural kinds, semanticization, transitional gradation, animal cognition

## Abstract

In this article, we explore various arguments against the traditional distinction between episodic and semantic memory based on the metaphysical phenomenon of transitional gradation. Transitional gradation occurs when two candidate kinds A and B grade into one another along a continuum according to their characteristic properties. We review two kinds of arguments—from the gradual semanticization of episodic memories as they are consolidated, and from the composition of episodic memories during storage and recall from semantic memories—that predict the proliferation of such transitional forms. We further explain why the distinction cannot be saved from the challenges of transitional gradation by appealing to distinct underlying memory structures and applying our perspective to the impasse over research into ‘episodic-like’ memory in non-human animals. On the whole, we recommend replacing the distinction with a dynamic life cycle of memory in which a variety of transitional forms will proliferate, and illustrate the utility of this perspective by tying together recent trends in animal episodic memory research and recommending productive future directions.

This article is part of the theme issue ‘Elements of episodic memory: lessons from 40 years of research’.

## 1. Introduction

A tree diagram enshrining the distinction between episodic and semantic memory (hereafter, the ‘E/S distinction’) has long been a fixture of textbooks in cognitive psychology and neuroscience. Most connected to the work of Tulving [1], the E/S distinction has achieved hegemonic status in psychology and neuroscience, though alternative views that proposed a continuum between various forms of memory have remained live empirical options throughout all these years (i.e. [2–8]).^1^ These continuum hypotheses have not received as much attention outside the subdiscipline of memory psychology—especially animal cognition, developmental cognition, and artificial intelligence, which have tended to treat Tulving’s view as monolithic. We here join other recent voices in arguing that more recent empirical and philosophical concerns have turned the tide in favour of the continuum views (e.g. [14–18]), especially by grounding the case on a firm philosophical foundation and by spelling out implications for these other subdisciplines in a way that we hope will illustrate the appeal of continuum views more widely.

Specifically, we systematize diverse continuum hypotheses and reframe the central question of this debate using tools from philosophy of science and especially the theory of natural kinds, which can help us proceed more fruitfully (for similar recent efforts, see [19–21]). In particular, the theory of natural kinds can clarify the relationship between representations, neural systems, and recollective experience, and how the E/S distinction stacks up against similar borderline disputes in other sciences. More specifically, we here argue that the E/S distinction does not mark out two distinct natural kinds because, like a variety of other distinctions in the life sciences, it exhibits the metaphysical phenomenon of transitional gradation. If we explore the full range of autobiographical memory phenomena in humans, we see that semantic and episodic memories display many systematic transitional forms in human psychology, and any attempt to carve a natural joint between those transitional forms would be arbitrary.

Even if this is conceded, however, defenders can retreat to a fallback position that justifies the E/S distinction in evolutionary terms. Specifically, they note that (non-human) animals have excellent semantic memory but poor or non-existent episodic memory, so animals constitute an ‘evolutionary dissociation’ between the two kinds of representation that justifies a non-arbitrary distinction in comparative psychology [22,23].^2^ To counter this fallback position, we must explain why animals cannot exhibit the same levels of success on episodic recollection tasks despite exhibiting impressive performance on semantic memory tasks. We conclude the article with a line of research designed to counter this rebuttal by arguing that animals are unable to excel on episodic recollection tasks because they lack temporal landmark words, but this might be addressed by experimental paradigms deploying non-linguistic temporal landmark cues. If these experiments are successful, it would undermine the conclusion that animals lack episodic memory entirely and complete a resilient argument against the E/S distinction. It would moreover, we think, ground a more productive approach to the study of animal episodic memory than the current stalemate that has developed over the phenomenological criteria for episodic memory recommended by some defenders of the E/S distinction.

This article is organized as follows. Section 1 reviews arguments against a natural kind distinction between episodic and semantic memory from semanticization and memory composition. In §2, we provide a firm metaphysical foundation for continuum arguments based on the transitional gradation of the underlying mechanisms supporting episodic and semantic memories. Section 3 addresses the rebuttal to our argument sketched above: that a natural kind distinction can be preserved in comparative psychology despite transitional gradation in humans, given that non-human animals have excellent semantic memory but poor or non-existent episodic memory. In response, we suggest that the challenges animals have displayed in exhibiting human-like forms of episodic recollection follow from computational difficulties of spatiotemporal indexing diverse memories when provided with impoverished recall cues. In §4, we briefly review studies of episodic memory and mental time travel in non-human animals that support this proposal, and end by suggesting a novel line of experimental research that might provide animals with the requisite spatiotemporal recall cues. In particular, we highlight the need for a compositional cue structure that can scaffold the kind of flexible spatiotemporal indexing at which humans excel.

## 2. Semanticization challenges to the episodic/semantic memory distinction

The simplest criterion for a distinction between episodic and semantic memory systems focuses on the representational content imputed to the memories—the ‘nature of the stored information’ [1]. Episodic memories are memories of events as perceived in one’s own personal past, subjectively contextualized by ‘its temporal–spatial relation to other experienced events’ [1, p. 388]. The attempt to operationalize this content-based criterion eventually led to the catchy mantra that the content of episodic memories could be identified as a representation of the ‘what–when–where’ information for personally experienced events [27–29].

Episodic memory theorists like Tulving have been aware from the beginning that a purely information-based criterion fails to neatly cleave episodic and semantic memory systems. In particular, semantic memory is by hypothesis flexible enough to represent the very same what–when–where information about the same events that would be encoded in any episodic memory. For example, the paradigmatic semantic memory that ‘Columbus sailed the ocean blue in 1492’ contains information about what happened, where it happened and when it happened—even in relation to other events. Indeed, these semantic memories about events could even be autobiographical, such as a semantic memory for an oft-recounted accident from early childhood that the subject herself cannot episodically recall in adulthood. Episodic memory theorists thus often rely additionally on a phenomenological criterion, noting that episodic rememberings often come with a subjective ‘sense of reliving’ that is missing from recollection of abstract facts.

Perhaps a more serious challenge to the distinction is posed by the phenomenon of semanticization (e.g. [14]; see also [9]). Semanticization involves a migration from episodically encoded and stored memories to semantically encoded and stored memories. Aronowitz (2023) cites two prominent theories of memory that entail semanticization: the complementary learning systems theory and the navigational theory. The complementary learning systems theory posits two different memory systems: the hippocampal system and the neocortical system, with a consolidation process. This process of consolidation entails gradual semanticization because the results of consolidation are semantically formatted memories stored in neocortical structures. The navigational theory proposes that just as spatial navigation proceeds gradually from egocentric reference classes to allocentric reference classes over repeated exposures, episodic memories gradually shift to decontextualized semantic memories over time and with repeated exposures. Both theories predict that normal memory function involves the gradual conversion of episodic into semantic memories. Memories midway through the process of abstraction may exhibit properties of both forms of memory—even partially autonoetic components, such as the ‘repisodic’ memories described by Neisser of a stereotyped, repeated event, such as a traditional family holiday dinner [30–32].

Gradual semanticization problematizes a sharp distinction between episodic and semantic memories. Aronowitz further articulates two of these challenges—the argument against content and the argument against function. If the depersonalization characteristic of semantic memory is achieved through semanticization, then the process requires an incremental shift from episodic content to semantic content, with a proliferation of transitional borderline cases in between. Now consider instead a different distinction based on the idea that episodic and semantic memories serve different functions: according to Aronowitz, a function-based approach will fail for similar reasons, for the semanticization story requires a tight link between episodic storage and retrieval and semantic storage and retrieval, and during semanticization transitional forms of memory will naturally play an admixture of two different functional roles.^3^

In addition to transitional pressure generated by the migration of memory from episodic to semantic forms, there is pressure in the opposite direction as well. What we call ‘the composition argument’ notes that episodic memories must be composed by binding together abstracted semantic memories. In particular, Renoult *et al.* [33] propose that an episodic memory is composed of ‘a conjunction of familiar concepts and episode-specific information (such as sensory and spatial context)’ [30, p. 1046]. By drawing on the trace transformation theory [34], Renoult *et al*. argue that episodic recall enlists various semanticized, depersonalized memories as well as perceptual and spatial representations that are bound together. Furthermore, the amount of perceptual detail and semantic facts in episodic recall can vary flexibly depending on current task demands (see also [17,18]). In short, the ‘what’, ‘when’ and ‘where’ components stored and reconstructed in an episodic binding could only be derived from semanticized abstract facts, and again, the theory predicts a proliferation of transitional forms in episodic recall’s routine and proper function.

Together, semanticization and composition entail a tight, dynamic interplay between episodic and semantic memories. Specifically, episodic memories are semanticized via consolidation, but, moreover, episodic retrieval involves activation and binding of various semanticized memories together with perceptual and spatial representations. The result is a cyclical challenge to the E/S distinction that recommends replacing a stark dichotomy with a richer life cycle of mnemonic representations, featuring numerous transitional forms: throughout the semanticization of episodic memories and throughout the composition of episodic memories from variously abstracted semantic, perceptual and spatial representations (see also [35]).

## 3. Accommodation failure in the distinction between episodic and semantic memory due to transitional gradation

To determine whether the challenges from semanticization and composition are fatal to the E/S distinction, it will help to review theories of natural kindhood from philosophy of science. A theory of natural kinds attempts to define a general criterion for deciding when a proposed scientific category is a useful target for scientific investigation. The earliest theories of natural kinds presupposed that kinds are united by microstructural essences that are present in all and only members of the kind and that explained their characteristic macroproperties—like the molecular structure H_2_O for the natural kind *water* or a particular genetic code for biological kinds such as *tiger* or *chimpanzee*. As microbiology advanced, however, it became obvious that there was no particular genetic code possessed by all and only members of a particular biological species—and indeed, a certain amount of random genetic variation on which natural selection could act is required by the ‘modern synthesis’ theory of evolution [36]. As a result, more resilient theories of kindhood were proposed that could accommodate borderline cases. These theories might tolerate the transitional cases of memory that we discussed in §2, so we need to proceed cautiously from the premise that transitional gradation proliferates in memory to the conclusion that the E/S distinction must be rejected.

The most popular recent account of kindhood for the life sciences is the homeostatic property cluster (HPC) account, most associated with the work of Boyd [37,38]. According to the HPC theory, natural kinds are useful for sciences to study because kind members share a set of properties that reliably and non-accidentally cluster owing to the operation of some underlying causal mechanism(s). Notably, however, this view does not require necessary and sufficient conditions for kind membership. The link to underlying mechanisms provides the view with additional resources to accommodate borderline cases: if the clustering of characteristic properties is caused by two distinct sets of underlying mechanisms, then we can maintain a principled and scientifically relevant distinction between the two categories even in the face of transitional gradation in their surface properties. For example, monarch butterflies (*Danaus plexippus*) and viceroy butterflies (*Limenitis archippus*) are distinct species despite appearing almost indistinguishable in terms of morphological properties, because the two species possess two different sets of underlying developmental mechanisms that produce and explain those properties, and they cannot interbreed. Mimicry explains why viceroys resemble monarchs, rather than a shared developmental process, evolutionary descent, and processes of interbreeding.

The present question is whether, given pervasive transitional gradation, there are non-arbitrary reasons to draw the borderlines of episodic memory approximately where Tulving drew them, specifically so as to exclude semantic memory and animal episodic-like memory. More generally, transitional gradation occurs when two candidate kinds A and B blur into one another along a continuum defined by their characteristic properties. Such gradation is systematic rather than accidental when it is not the result of outside influence or unlikely perturbations in an otherwise stable system, but rather when the same causal processes or mechanisms that produce the characteristic properties of both A and B ensure that borderline admixtures of those properties will systematically proliferate. In such cases, the problematic variation cannot be explained or abstracted away, lest we frustrate the search for the mechanisms that explain the clustering of A properties and B properties in the first place [36]. Boyd calls such cases a failure of ‘accommodation’ between our proposed taxonomy and the underlying structure of the world.

Transitional gradation is problematic for sciences still developing their core taxonomies because it can take significant investigation to uncover the degree of transitional gradation, whether the gradation is systematic or due to random noise (such as genetic drift) and when to abandon further attempts to save a distinction through revision of its precise boundaries as unproductive. Each failure in accommodation can initially be written off as an early draft of the kind’s characteristic properties that can be saved by revising the putative list of the kind’s characteristic properties. The problem with systematic transitional gradation, however, is that the fit between our taxonomy and the world never improves with revision; we repeatedly reach out with revised programmatic definitions of A and B for better contact with nature’s ‘joints’ but are repeatedly embarrassed by the discovery of yet more smooth continua. When the source of transitional gradation is deep explanatory structure, it is inescapable—because the same explanatory structures produce the properties of the borderline cases, too. In such cases, any line drawn—even a fuzzy one—would ultimately be arbitrary, in the sense that we could not justify why it should be drawn at that location on the continuum rather than anywhere else. This intractability amounts to a deal-breaker for any distinction between A and B; and the best outcome we can hope for would be a rejection of the original distinction and accommodation with a new lumped superkind category, AB [39].

**Figure 1.**
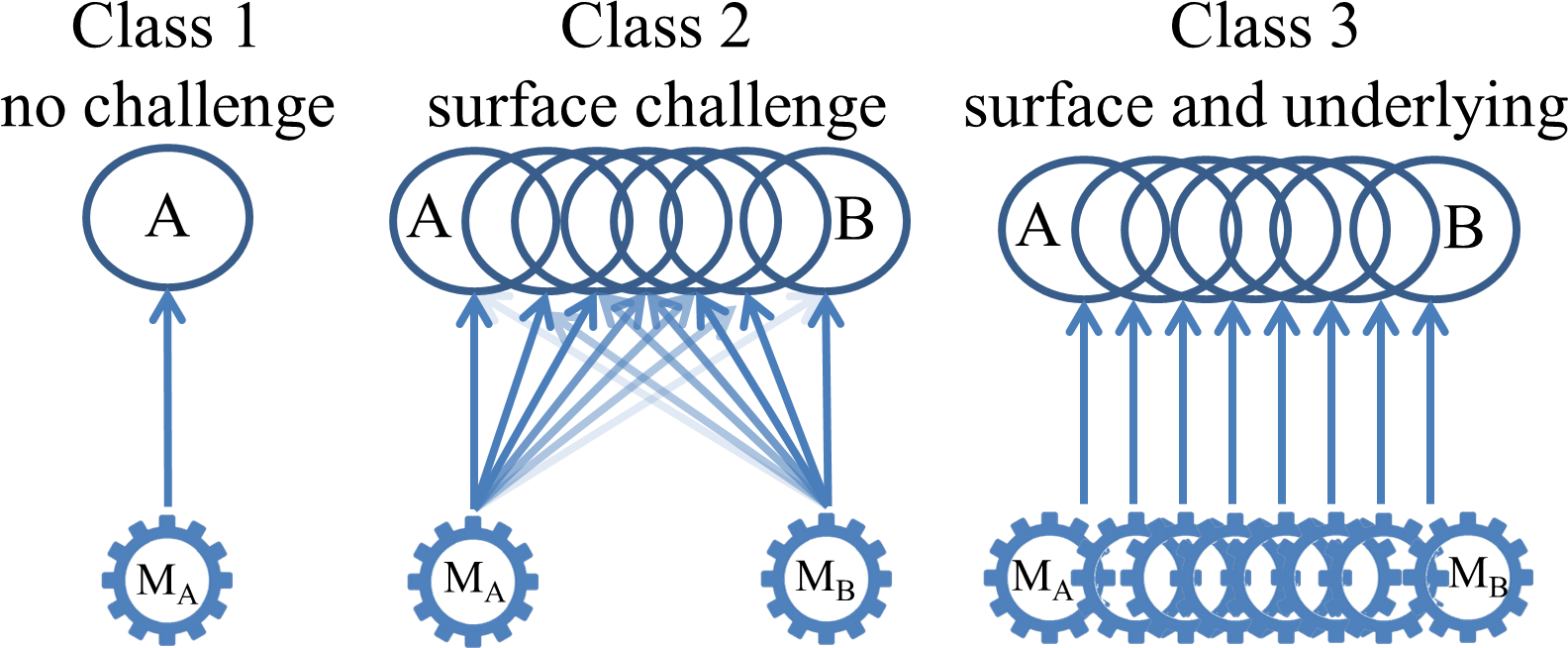
Levels of increasingly difficult challenge from transitional gradation to distinction between A and B. Circles labelled with A and B indicate clusters of properties in programmatic definitions (in this case, properties of memory representations or their recollection): gears indicate underlying mechanisms that produce those properties, as might be found in corresponding explanatory definitions. Arrows indicate causal relationships. Increasingly faded lines indicate a lesser degree of causal participation in the relevant exemplars [39].

Consider now how the challenges coming from transitional gradation can be categorized in terms of three different classes of increasingly weak accommodation between criteria for episodic memory and underlying causal mechanisms. In the simplest case—a class I accommodation (see figure 1)—there is a nice fit between surface properties and underlying causal mechanisms that cause them to non-accidentally cluster (e.g. with water and H_2_O—see figure 1). In Tulving’s later writings, he can be read as conceding that two distinct class I accommodations for episodic and semantic memories are untenable but that we might yet have a class II accommodation (as with the monarchs and viceroys), where we can tease apart the influence of distinct underlying memory systems in two different explanatory definitions. The question of whether transitional gradation sinks the distinction can thus be recast in terms of different degrees of accommodation failure, depending upon whether transitional gradation is found only in the distribution of surface properties captured in programmatic definitions or whether it also reaches down to a continuum at the level of underlying neural mechanisms. Should there also be transitional gradation at the level of underlying mechanisms, then we should be pushed into a class III challenge, where the best approach accepts a new lumped superkind—and the E/S distinction, if retained at all, could only serve heuristic purposes.

Tulving’s own thoughts on the distinction can be fitted to the apparatus of Boyd’s HPC theory and these classes of challenge, for he at times appealed to underlying mechanisms to bolster the distinction. For example, he suggested that we should not ask ‘how does a systems theorist unambiguously identify a particular memory as being in one system or the other’ ([40, p. 233], quoted by [41, p. 5]), but instead ‘whether it is possible for damage to the brain to occur in such a way that [episodic memory] is deleteriously affected while other kinds of memory are not, or are less affected’ [41, p. 12]. Although such double dissociations have been considered the gold standard for drawing modular conclusions about brain functions, there are reasons to be sceptical. In particular, it has been argued that double dissociations do not tell us much unless modularity is antecedently assumed [42]. Reasoning from double dissociations to distinct brain modules requires ‘pure cases’: lesions that disable all and only some critical causal component of a particular mental function and not components of another. Van Orden *et al*. point out that there is no theory-neutral way to know whether a particular lesion is a pure case (i.e. in this case, disabling episodic memory but leaving semantic memory entirely intact, and *vice versa*) and so cannot be used to arbitrate taxonomic disputes about kinds of memory [43]. In the case of the E/S distinction, there have long been doubts that particular lesion patients count as pure cases of dissociation, as supposedly pure episodic lesion patients also show subtle semantic deficits (e.g. [6]) and patients with semantic dementia also show episodic-related deficits (e.g. [44]). Moreover, other theories here can explain lesion-induced deficits in other ways: for example, by suggesting that what is disabled in cases of MTL lesions is not episodic memory *per se*, but rather an entry pathway to both episodic memory and (via semanticization) semantic memory, or the constructive system required for both episodic recollection and imaginative exploration of events in semantic memory. Wais *et al.* [45] similarly argue against using double dissociations in this case by arguing that the remember/know probes used here track degrees of familiarity with both episodic and semantic memories, and so lack the needed conceptual purity (see also [25]). Thus, the present question cannot be settled by claiming that lesion studies recommend a class II accommodation for the distinction between episodic and semantic memories.

We here thus argue that the E/S distinction suffers from a class III accommodation failure resulting from pervasive transitional gradation, even at the level of underlying mechanisms. In particular, we argue that episodic memory and mental time travel abilities are exaptations of medial temporal lobe structures (especially the hippocampus and entorhinal cortex) possessing a more general function of arranging stimuli along ordinal monotonic dimensions and allowing agents to flexibly navigate around those representational spaces. For example, in cognitive mapping, agents frequently need to re-route to their goal when familiar routes are obstructed. The ability to flexibly re-route is subserved by spatial monotonic orderings of locations and landmarks in an integrated map-like representation. In episodic memory, the ability to mentally travel back in time to previously experienced events is subserved by the same system—one that monotonically orders events by temporal indices. Moreover, flexibly navigating this space of event representations means that the agent can target different memorial contents in different contexts with the same cue. Later, we will argue that this ability is precisely what explains the rift between humans and animals because human-like flexibility is (at least) partially explained by compositional linguistic abilities.

This approach connects the universally accepted view that the medial temporal lobes are at least a system for encoding spatial relations to a rich history of viewing abstract conceptual knowledge as organized according to similarity relations in multidimensional space [46–48]. Here, convex regions of space correspond to concepts (in any domain), and the distances between regions represent semantic similarity, with greater cosine distances representing greater semantic dissimilarity [49]. Neural mechanisms that were adapted to representing external spatial relations in the environment were exapted to represent a variety of other more abstract relations in semantic space as well (see also [10,50,51]). The functional flexibility of such encoding schemes is further bolstered by Gazes *et al.* [52], who summarize behavioural and neurobiological evidence for a common domain-general magnitude representation system that responds to (at least) physical magnitudes, space, time and dominance relations. Importantly, because of their shared underlying ordinal features, these magnitude representations can be extended to learned orderings and sequences (enabling limited degrees of transitive inference across a variety of domains).

Focusing on spatial representation, the most popular account of these mechanisms in mammals involves place and grid cell interactions in the medial temporal lobes to construct map-like representations of the environment [53]. In (environmental) spatial representation, place cells respond to locations and bind together various snapshots taken from different egocentric frames, and grid cells represent the locations of these bundles with respect to one another in spatial dimensions by linking them to a spatially organized array. Importantly, visible spatial landmarks play an important role in establishing these links. The grids are anchored by landmarks, and the same landmarks are visible from different egocentric viewpoints. As Gazes *et al*. [52] note, much evidence suggests that the place and grid cell systems are redeployed to represent and reason about other monotonically ordered domains, such as time [54–57]. For example, there is evidence that entorhinal ‘ramping cells’ have firing rates that track temporal order of events [58,59].^4^ Moreover, some psychologists have argued that ‘temporal landmarks’ play a similar role to spatial landmarks in anchoring a system of temporal representation in children [60,61]. Even broader functions for grid coding have also recently been studied, such as ‘social place cells’ for abstract position in a social hierarchy [62]. This evidence all fits broadly into more general trends in cognitive ontology that suggest that brain circuits are routinely re-used for many different domain-specific functions [63].

To summarize, empirical evidence suggests that neural mechanisms that were thought to be dedicated to spatial navigation or episodic memory are in fact implicated in routine acts of semantic memory recall and inference, and indeed in relational representation and navigation more generally (see [64] for a review). In particular, we think recent neuroscientific evidence supports the view that mental time travel is subserved by a domain-general relational ordering system that deploys place and grid cells to construct and navigate map-like representations that are arranged along ordinal and monotonic dimensions in a wide variety of domains. If this is right, then the mechanisms underlying episodic and semantic memories are dynamically intertwined in their routine operations and continuously grade into one another at the mechanistic level (i.e. suffer from a class III accommodation failure), and surface transitional gradation cannot be explained away by appealing to distinctions in underlying mechanisms. Landmarks (be they spatial or temporal) notably play an integral role in navigating these representational spaces because they help link together various points in these spaces. In §4, we turn our attention to the stalemate in episodic memory research in non-human animals and appeal to this role of navigational landmarks to suggest new directions for research.

## 4. The argument from animals

At this point, defenders of the E/S distinction might point out that non-human animals, as a matter of empirical fact, exhibit deficient (or non-existent) episodic abilities but have excellent semantic memory. This might justify an evolutionary dissociation, thereby securing an E/S distinction on evolutionary grounds. To rebut this position, we need to explain why animals fail to show human-like episodic abilities despite having excellent semantic memory. However, in order to assess the merits of such a fallback position, it is worth briefly placing the position in the larger context of animal episodic memory research. At the end of the article, we sketch a new line of research designed to counter this fallback position. In particular, we propose that experimental paradigms involving non-linguistic, compositional temporal landmark cues could scaffold animal episodic abilities to better approximate human-like episodic abilities.

To provide background on the animal research, the landscape of episodic memory research was shaken somewhat in 1998, when Clayton & Dickinson [27] provided evidence that scrub jays have episodic-like abilities. These well known experiments appeared to show that scrub jays could flexibly deploy ‘what–when–where’ information in recovering cached food items. However, as mentioned earlier, Tulving [22] and other ‘human uniqueness’ theorists (see, e.g. [23] and [65]) responded to these studies by further emphasizing the subjective, experiential aspects of recollection, especially autonoetic awareness, or the subjective sense of reliving [41,66]. With the introduction of an empirically intractable phenomenological criterion on episodic abilities, many animal researchers continue using a content-based criterion, while worrying that success on tasks shows mere ‘episodic-like’ abilities (see [67] for review and critical discussion).

### (a) Diagnosing the animal research stalemate

As a result, a stalemate has developed in research on episodic memory in animals. One side emphasizes a content-based criterion, whereas the other prefers a phenomenological criterion. The obvious problem is that, in the absence of language, we have only very indirect access to animal phenomenology, so sceptics will interpret all findings in animal memory as merely ‘episodic-like’. Part of the problem here is that these criteria are assumed to cleave episodic and semantic memories into distinct kinds, a view we have argued above is flawed on independent grounds. However, our view can be taken to show that there is something right on both sides of the stalemate. It also provides a route to counter the evolutionary dissociation position outlined at the beginning of the article.

We agree with proponents of animal episodic memory research that at least some animals possess the same basic mechanisms we do for episodic memory, but we also agree with the sceptics that animals will likely exhibit only below-human-level episodic abilities—though, as we shall see, this is not because they fail a phenomenological criterion. Instead, we think distinctively human ability is afforded by compositional linguistic abilities to organize, navigate and address fine-grained, qualitatively ambiguous but monotonically ordered temporal representations.

The idea that episodic abilities in humans and animals differ only in degree rather than kind might initially seem surprising, but this surprise can be ameliorated by looking closer at how human children gradually acquire human-like degrees of flexibility in cognitive development. This developmental story emphasizes children’s increasing use of temporal landmarks to represent and reason about temporal relations, especially by acquiring and deploying temporal landmark words. After an especially memorable life transition, children might start ordering other events around that landmark using stable, unambiguous indexing words (e.g. ‘before I started preschool’, ‘after we moved to the new house’). Children can then order these events with respect to a few indices sequenced by major events specified according to more absolute temporal references (e.g. ‘when I was 4’, ‘when I was 5’). Only later, after learning more ordered sequences of temporal landmarks, can they progress to a series of hierarchically nested indices that enable the level of fine-grained, monotonically ordered temporal representation that characterizes adult human cognition (times of the day, days of the week, months of the year, and so on [68]).

It is this final stage of development where temporal landmark words can be used to flexibly index specific events in an unambiguous way (‘the dinner last Thursday’, ‘the parade next month’). This ability is partially afforded by the compositionality of language. ‘Thursday’ on its own, for example, conventionally picks out the very next Thursday; however, compositionally adding the modifier ‘last’ alters the reference to the previous Thursday. Combining other labels and modifiers for various fine-grained temporal indices, we can refer to more and more specific times and events (e.g. ‘the sunset we saw three nights ago’, ‘the colloquium dinner last April’). Animals’ diminished episodic flexibility can thus be explained by the practical problem of navigating and addressing temporal dimensions without temporal language. Unlike spatial dimensions, previous or future locations in time cannot be physically revisited to refresh or disambiguate reference; unique events are experienced only once, and without language can only be indexed by their perceivable features, which will often be ambiguously associated with many other distinct events located at different temporal indices.

We thus argue that that the real difference between human and animal episodic abilities is grounded in the flexibility that each exhibits in mnemonic recall and inference. That is, humans flexibly navigate monotonically ordered event representations such that they can target different memorial contents in different contexts with the same cue (when combined with others). Furthermore, human-like flexibility is afforded by compositional linguistic abilities. In particular, the ability to compositionally combine temporal landmarking words allows humans to make fine-grained, unambiguous addresses in representational space.

This view predicts the fact noted earlier that patients with semantic dementia or semantic memory deficits should be deficient in flexible episodic abilities. Irish *et al.* [44] tested this prediction in semantic dementia and Alzheimer’s patients. The authors tested these patients’ episodic recall and episodic future thinking abilities. They found that patients with semantic dementia had relatively preserved episodic recall for *recent* past events, but deficits in episodic future thinking. Alzheimer’s patients had paired deficits for episodic recall and episodic future thinking. This finding (among others, see [69,70]) suggests that semantic memory, and, in particular, linguistic knowledge is necessary for flexible episodic abilities. Crucially, our claim is *not* that compositional linguistic abilities are necessary for episodic abilities generally. Rather, human-like flexibility in navigating and addressing monotonically ordered event representations in a fine-grained, context-sensitive way requires compositional linguistic abilities.

This view recommends a different approach to empirical investigation of episodic-like abilities in animals. In particular, we should emphasize experiments that provide animals with specific and compositional memory cues in the appropriate way. In particular, recall cues need to be unambiguous, but flexible enough to index different contents. Although it is controversial whether non-human animals possess compositional linguistic representational abilities at all, some recent work has shown that non-linguistic representational formats might compose, especially, map-like representations [71–74]. In §5, we will review a few empirical case studies on non-human animal episodic recall and mental time travel, using our recommendations to diagnose animals’ lack of human-level ability. We suggest that viewing these experiments as a trend in the right direction suggests a coherent new direction for animal episodic memory research.

## 5. Case studies

If our analysis is on the right track, then non-human animals should be able to engage in mental time travel given appropriate indexing cues. In humans, flexible cues are provided through natural language in the form of temporal landmarking terms; but without access to such language, non-human animals must rely on scarce environmental cues for addressing. The more common the cue in the organism’s environment, the greater the danger that unique indexing will fail and memories of multiple events will be simultaneously activated and conflated. In what follows, we briefly review three case studies testing for episodic memory and mental time travel in non-human animals, highlighting the role played by environmental cues in recall, and the way that cues may or may not meet the challenges of unambiguous memory addressing.

### (a) Replay of episodic memories in the rat

Panoz-Brown *et al.* [75] tested for episodic replay of sequences of unique events in rats. The experimenters placed a rat into an encoding context. The encoding context was an open-field arena that was square with 12 equidistant food holes arranged along the walls. For each encoding, the food holes were filled with trial-unique odourant cups (5–12 odours). After encoding, the rat was moved to the first of two memory assessment contexts. Both of the assessment contexts differed in size, shape and colour. In the first, the rats were rewarded for choosing the second-to-last odourant from the encoding list. In the second assessment context, the rats were rewarded for choosing the fourth-to-last odourant. The authors report that optimal performance requires selection of the second-to-last and fourth-to-last odours from the encoding list when in the appropriate memory assessment context, but rejection of odours previously encountered in different ordinal positions. Hence, performance requires memory of the order of events from encoding. The rats performed above chance and with accuracy across the experiments (>80% correct).

For each memory assessment task, the rats were placed into different arenas. For example, the first arena, where the rats had to select the second-to-last odour from the encoding list, was circular, white and had 18 food holes arranged in two concentric circles. The second arena, where the rats had to select the fourth-to-last odour, was also circular, but had transparent walls and three concentric circles on the floor alternating in colour (black, white, black). Hence, the cues to select either the second-to-last or fourth-to-last odour from the encoding list were given by the differences in the arenas. The combination of environmental differences provides unambiguous recall cues but little flexibility to index different contents. Compare with the case of temporal landmarking terms in natural language: ‘last Tuesday’ is unambiguous with respect to time frame, and it can be redeployed to index different contents. This begins to show that rats might have some ability to combine cues flexibly to index different events, though it would be difficult to scale this design up to human-level flexibility given the spatial constraints of the task.

### (b) Memory for distant past events in chimpanzees and orangutans

Another experiment assessed primates’ ability to combine cues acquired over longer temporal distances in a few-shot paradigm that in humans would likely implicate episodic memory. Martin-Ordas *et al.* [76] tested for episodic memory in chimpanzees and orangutans. Experiment 1 tested for episodic memory of a tool-finding event that happened four times 3 years earlier. Experiment 2 tested for episodic memory of a unique tool-finding event that happened two weeks earlier. For experiment 1, the apes observed an experimenter hiding two different tools in two locations. In order to receive a reward, the apes had to remember where the useful tool was hidden, retrieve it, and use it. The apes were exposed to this set of events four times, but then 3 years later, the apes were exposed to the food reward task. Here, the apes had to use a combination of environmental cues in order to retrieve their memory of where the useful tool was hidden. For example, the testing room, the experimental set-up and the experimenter were the same in the initial four trial presentations of the two events and the food reward task 3 years later.

Martin-Ordas *et al*. [76] note that it remains to be seen whether the apes would be successful in retrieving event memories after just one exposure and be able to distinguish them from other similar events. In experiment 2, the apes were shown a see-saw task and then observed an experimenter hide a tool in one of two locations. After a short interval, the apes were allowed to retrieve the tool. This was the initial encoding phase of the experiment. Two weeks after this phase, the apes were brought back to the same room and shown the see-saw task again, but not shown the experimenter hiding the tool. The apes were then allowed to find the tool and solve the task.

Results of experiment 1 showed that four of the apes retrieved the correct tool immediately and used it, while six retrieved the wrong tool, rejected it, and then retrieved the correct tool. For experiment 2, seven out of nine apes went to the correct location first. The other two went to the wrong location first and then went to the correct location. The authors conclude that the apes have recall for general events that happened 3 years ago as well as recall for unique events that happened two weeks earlier.

Martin-Ordas *et al*. argue that ‘in order for the cue combination to activate the relevant memory, subjects had to be able to bind these elements together and ignore a number of irrelevant associative links’ [76, p. 1438]. Again, however, constraints on the type of non-linguistic cues prevent this facility from scaling up to human-like levels of performance. Each cue on its own was insufficient to target the relevant memory because all cues were individually associated with distinct prior experimental contexts. This suggests that, although the apes were successful in the tasks (at least in part) because the combination of environmental cues was highly discriminating, the cues are nonetheless inflexible. Hence, they could not be redeployed to index different contents like human temporal landmark words.

### (c) Mental representation and episodic-like memory of own actions in dogs

Another experiment explored whether dogs could learn linguistic commands that had a flexible temporal indexing structure. Specifically, Fugazza *et al.* [77] tested for episodic recall of dogs’ own actions using unexpected testing after incidental encoding. In this study, Fugazza *et al.* [77] asked whether dogs could spontaneously recall their own actions and reproduce them without commands to perform a specific action. The owner of the dog sat on a bench or sofa in the dog’s familiar environment. While the dog roamed about freely, the owner ignored him/her. As soon as the dog performed an identifiable action (e.g. lay down or jumped on the sofa), the owner called the dog over and gave the ‘repeat’ command without training phases and with a single trial (over various time delays between action and command). With shorter delays, 70% of the dogs could successfully repeat a variety of actions in a single trial.

The recall cue, in this case, was the verbal ‘repeat’ command given by the owners. When there is no delay between the target action and the repeat command, the cue is fairly unambiguous. However, as the delay is increased, the experimenters observed a drop in performance. One potential explanation is that ambiguity increases proportionally to increases in delay. Nonetheless, the repeat command is (in principle) flexible in a way that cues in the previous two experiments were not, because it can be used to index a variety of events by temporal distance.

## 6. Conclusion and future directions

Given the studies just reviewed, we are sceptical that the ‘evolutionary dissociation’ position—that humans uniquely possess underlying neural mechanisms for episodic memory and recall—is tenable. We have argued that animals have deficient episodic abilities owing to their lack of unambiguous compositional temporal landmarking language. We argued that such language affords flexible, fine-grained addressing of memorial contents in representational space. Our arguments suggest that we should explore further forms of flexibility that animals might exhibit on episodic-like tasks by eliciting different memories with a flexible combination of temporal indexing cues, especially with animals we suspect can learn to respond to abstract symbolic or linguistic cues. Thompson & Oden’s [78] study of higher- order relational concept learning in language-naive chimpanzees suggests a model for this research. Thompson & Oden presented chimpanzees with a relational match-to-sample task with pairs of shapes by training chimpanzees to associate coloured tokens with abstract sameness and difference relations. Token-trained (but not naive) chimpanzees were successful in identifying sameness and difference in novel sets of stimuli. The explanation for this performance advantage, according to the authors, is that ‘external tokens functioned like words, providing the animals with concrete icons for computational and encoding processes involving abstract propositional representations’ [78, p. 382].

We propose adapting this symbol-learning paradigm to test for episodic abilities in non-human animals. We predict that insofar as animals can be successfully trained to associate various target events with the particular tokens, they will be able to recall those specific events when cued with those particular tokens. One way to test recall would be to utilize eye-tracking technology. In a comparative study, Kano & Tomonaga [79] showed striking similarities between chimpanzee and human eye movements. Indeed, it was recently shown that chimpanzees have a strong looking preference for former, distant (<26 yr) groupmates [80]. Utilizing eye-tracking, experimenters could cue the chimpanzees with one temporal indexing token from training and then expose the chimpanzees to two previously shown events (one being the target event represented by the token cue). For example, experimenters could cue the chimpanzee with a blue circle token (representing one temporal index) and then expose the chimpanzee to pictures of two events with similar perceptual properties but different temporal indices. If the chimpanzee looks longer at the temporally matching event, then this would count as success. Of course, stimulus properties of the cue alone might be confounded with associative learning on the events from training, so the real test is whether chimpanzees can distinguish between two events using novel combinations of compositional cue structure.

In another experimental design (inspired by [77]), we imagine training animals (e.g. chimpanzees) to associate their own actions from different domains with particular tokens. For example, we might associate blue tokens with problem-solving events (PSEs) and red tokens with eating events (EEs). The shape of the tokens could then be associated with different temporal indices within those categories. For example, an earlier event PSE-A could be associated with a blue circle, EE-A with a red circle and EE-B with a red square. Similar to Fugazza *et al*. [77], experimenters could then test for recall by providing chimpanzees with a token novel compositional structure to see whether they could flexibly index a particular PSE. For example, if cued (for the first time) with a blue square, the chimpanzee with flexible episodic abilities should repeat the action in PSE-B, and not in PSA-A or EE-B.

In summary, this article contrasted a popular dichotomous approach to episodic and semantic memories with a more dynamic approach based on a transitional life cycle of mnemonic representations. We hope to have illustrated that, rather than serving only critical aims, this philosophically grounded perspective can provide a fruitful foundation for future research on episodic ‘memory’ and mental time travel across several different subdisciplines of research.

## Data Availability

This article has no additional data.

## Footnotes

^1^
There are also authors who express a scepticism about a categorical distinction, without explicitly committing to a continuum view (e.g. [9–13]).

^2^
This approach tends to characterize episodic memory in terms of a distinctive kind of conscious experience during recollection that is purportedly uniquely human, so-called ‘autonoesis’. In humans, the phenomenal experience of remembering does offer apparently sharp boundaries in recollective experiences, but we argue that these differences are best explained in terms of an error theory—i.e. a theory that explains why subjects would mistakenly suppose there are two distinct kinds of memory accessed [24–26]. This error theory, we believe, makes better sense of the available data on humans while simultaneously offering ecumenical guidance on the apparent impasse in assessing the presence of episodic phenomenology in non-human animals.

^3^
Similar worries about functional indeterminacy are pressed by Robins [15] against even more recent defences of functional defences of the distinction by Khalidi [21].

^4^
Aghajan *et al.* [58] suggest that ramping cells are grid-like cells in that their activity constitutes a fourth dimension (time) in topological representations of the environment.
